# The Role of Adenine Nucleotide Translocase in the Mitochondrial Permeability Transition

**DOI:** 10.3390/cells9122686

**Published:** 2020-12-15

**Authors:** Nickolay Brustovetsky

**Affiliations:** Department of Pharmacology and Toxicology, Stark Neurosciences Research Institute, Indiana University School of Medicine, Indianapolis, IN 46202, USA; nbrous@iu.edu; Tel.: +317-278-9229; Fax: +317-274-7714

**Keywords:** calcium, permeability transition pore, adenine nucleotide translocase, cyclophilin D, F_0_F_1_-ATP synthase

## Abstract

The mitochondrial permeability transition, a Ca^2+^-induced significant increase in permeability of the inner mitochondrial membrane, plays an important role in various pathologies. The mitochondrial permeability transition is caused by induction of the permeability transition pore (PTP). Despite significant effort, the molecular composition of the PTP is not completely clear and remains an area of hot debate. The Ca^2+^-modified adenine nucleotide translocase (ANT) and F_0_F_1_ ATP synthase are the major contenders for the role of pore in the PTP. This paper briefly overviews experimental results focusing on the role of ANT in the mitochondrial permeability transition and proposes that multiple molecular entities might be responsible for the conductance pathway of the PTP. Consequently, the term PTP cannot be applied to a single specific protein such as ANT or a protein complex such as F_0_F_1_ ATP synthase, but rather should comprise a variety of potential contributors to increased permeability of the inner mitochondrial membrane.

## 1. The Permeability Transition Pore: Introduction

Mitochondrial Ca^2+^ uptake is one of the major mechanisms to lower cytosolic Ca^2+^ under conditions of Ca^2+^ overflow in the cytosol. Mitochondria possess Ca^2+^ channels in the inner membrane (historically called “mitochondrial calcium uniporter”) that mediate Ca^2+^ influx into the organelles driven by high membrane potential across the inner mitochondrial membrane [1,2,3,4]. This allows mitochondria to accumulate significant amounts of Ca^2+^. This large Ca^2+^ uptake capacity of mitochondria is enabled by the formation of biologically inactive Ca-phosphate precipitates in the mitochondrial matrix [5]. Although mitochondria can accumulate substantial amounts of Ca^2+^, mitochondrial Ca^2+^ uptake capacity is finite, and mitochondrial Ca^2+^ overload comes with a high price. Excessive Ca^2+^ accumulation by mitochondria can cause mitochondrial damage manifested in the induction of the mitochondrial permeability transition pore (PTP).

Inhibition of the PTP augments Ca^2+^ accumulation in mitochondria, stabilizes mitochondrial membrane potential, and defers Ca^2+^ dysregulation. Induction of the PTP, on the other hand, leads to mitochondrial depolarization, inhibition of ATP synthesis and Ca^2+^ uptake, mitochondrial swelling, and potentially the rupture of the outer mitochondrial membrane. The latter may result in the release of mitochondrial apoptogenic proteins such as cytochrome *c*, Smac/DIABLO, endonuclease G, and AIF. It is not surprising, therefore, that studies of the PTP attract significant attention. Despite significant effort, the exact molecular composition of the PTP is still a matter of hot debate. This review will primarily be focused on the role of the adenine nucleotide translocase (ANT) in the mitochondrial permeability transition.

## 2. Early Studies of the PTP

Early observations of the PTP manifestations were made in the 1950s and 60s (for review, see Reference [6]). However, the concept of the mitochondrial permeability transition was expressed and introduced by Hunter and Haworth only in the late 1970s [7,8,9,10]. In these papers, Hunter and Haworth used the term “Ca^2+^-induced membrane transition” in mitochondria to signify the large increase in inner membrane permeability and the key role of Ca^2+^ in triggering this phenomenon [8]. In their seminal work, Hunter and Haworth demonstrated that the Ca^2+^-induced membrane transition in mitochondria leads to mitochondrial uncoupling, the release of previously accumulated Ca^2+^, and mitochondrial swelling. The authors described major fundamental properties of the permeability transition, such as the requirement for Ca^2+^ entry into mitochondria, protection by reduced NADH and mitochondrial energization (membrane polarization), facilitation by membrane depolarization, and inhibition by H^+^, Mg^2+^, and other cations [8,9].

One of the early hypotheses concerning the mechanism of the Ca^2+^-induced permeability transition postulated that the increase in inner membrane permeability occurs as a result of activation of mitochondrial Ca^2+^-dependent phospholipase A_2_ and accumulation of free fatty acids and lysophospholipids that disrupt the phospholipid bilayer [11,12]. However, the discovery that the immunosuppressant cyclosporin A (CsA) suppresses the permeability transition [13,14], but does not inhibit mitochondrial phospholipase A_2_ [15], suggested that induction/activation of a proteinaceous pore in the inner mitochondrial membrane is most likely responsible for the permeability transition. Since then, the hypothesis that the PTP is a proteinaceous pore has dominated the field. Nevertheless, under conditions of massive Ca^2+^ accumulation by mitochondria, CsA inhibits PTP only transiently, followed by CsA-insensitive permeability transition, in which activated phospholipase A_2_ may play an important role [16]. Moreover, palmitate, a free fatty acid that is predominately generated due to phospholipases A_2_ activity, can induce a CsA-insensitive pore in the inner mitochondrial membrane [17], thereby contributing to Ca^2+^-induced mitochondrial damage beyond the classic CsA-sensitive PTP.

## 3. The Roots of ANT Hypothesis

In early studies, Hunter and Haworth (1979–80) described ADP’s inhibitory effect on the permeability transition, showing that it acted on the matrix side of the inner mitochondrial membrane (IMM). They also demonstrated the opposite effects of atractyloside and bongkrekic acid [8,18], ANT inhibitors that stabilize ANT in *c*- (nucleotide-binding site facing the cytosol) and *m*- (nucleotide-binding site facing the mitochondrial matrix) conformational states, respectively [19]. In ADP-depleted mitochondria, atractyloside stimulated, whereas bongkrekic acid antagonized, the permeability transition [8]. This suggested the involvement of ANT in the permeability transition. Consistent with this, an earlier study by Asimakis and Sordahl (1977) showed that atractyloside and an endogenous ANT inhibitor, palmitoyl-coenzyme A, induced the release of previously accumulated Ca^2+^ from isolated cardiac mitochondria [20], evidently due to induction of the PTP.

Shortly after that, Panov et al. (1980) showed that ADP, in a carboxyatractyloside-sensitive manner, inhibited H^+^ and K^+^ permeability in isolated liver mitochondria [21]. This was interpreted as evidence for ANT being a pathway for passive H^+^ and K^+^ transport across the inner mitochondrial membrane. It has also been speculated that ANT operates as a gated pore [21]. This hypothesis is supported by the ANT’s structural model that was proposed soon after and was suggested to operate as a gated pore [22]. Toninello et al. (1983) showed that ADP and ATP protect mitochondria from Ca^2+^-induced sustained membrane depolarization [23]. Bongkrekic acid also protected against depolarization, whereas atractyloside accelerated it. Interestingly, bongkrekic acid facilitated the protective effect of adenine nucleotides, while atractyloside abolished this protection. The authors agreed with the idea of Panov et al. (1980) that ANT, operating as a gating pore, might be responsible for the Ca^2+^-induced increase in membrane permeability [23]. Le Quoc and Le Quoc (1988) showed that carboxyatractyloside, palmitoyl coenzyme A, and pyridoxal phosphate, membrane-impermeant inhibitors of ANT, stimulated PTP induction, whereas bongkrekic acid and ADP hindered the permeability transition [24]. These authors also provided evidence suggesting that oxidation of sulfhydryl groups linked to oxidation of mitochondrial pyridine nucleotides influenced ANT’s conformation, stabilizing it in *c*-conformation [25]. The latter was linked to the increased probability of the permeability transition.

## 4. Mitochondrial Cyclophilin D and Its Interaction with ANT

In the late 1980s, it was found that CsA binds cyclophilin, a peptidyl-prolyl cis-trans isomerase involved in the folding of different proteins [26,27]. Later, it was shown that mammalian mitochondria have a distinct cyclophilin isoform, cyclophilin D (CyD) [28]. Currently, CyD is the only regulatory component of the PTP considered to be proven. Experiments with CyD-knockout mice showed that genetic ablation of CyD suppresses the permeability transition and increases Ca^2+^ uptake capacity in mitochondria by two to three-fold [29,30,31,32]. Based on these studies, it was postulated that CyD sensitizes the pore to Ca^2+^ [32]. Importantly, oxidative stress facilitates the recruitment of mitochondrial CyD to the inner membrane and promotes the permeability transition [33].

Consequently, the effect of CsA on the permeability transition was shown to be mediated by CsA interaction with CyD [34,35]. At the same time, studies of the ANT involvement in induction of the PTP continued. In 1990, Halestrap and Davidson showed that Ca^2+^-induced swelling of heart mitochondria was inhibited by ADP and bongkrekic acid, and the effect of ADP was reversed by carboxyatractyloside, a more potent derivative of atractyloside [34]. The authors suggested that Ca^2+^ interacts with ANT when it is in *c*-conformation and proposed the model in which mitochondrial CyD interacts with ANT in the presence of Ca^2+^, leading to the permeability transition. Later, Andrew Halestrap’s group experimentally proved this hypothesis by demonstrating mitochondrial CyD binding to ANT in the inner mitochondrial membrane and purified ANT [36]. However, these authors failed to find evidence for CyD binding to mitochondrial porin. Later, James Lechleiter’s group (2002) also demonstrated the binding of CyD and ANT [37].

In contrast to Halestrap’s findings, Martin Crompton’s group (1998) reported that CyD binds to a complex of mitochondrial porin (voltage-dependent anion channel, VDAC) and ANT to form the PTP [38]. The authors purified and reconstituted porin, ANT, and the glutathione S-transferase/CyD fusion protein into phosphatidylcholine liposomes loaded with fluorescein. These proteoliposomes were permeabilized by Ca^2+^ plus P_i_, and CsA inhibited this permeabilization. Thus, the authors concluded that the PTP includes porin, ANT, and CyD, and that it is possibly formed at the contact sites between the outer and the inner mitochondrial membranes.

## 5. Investigation of ANT as a Pore in Reconstituted Systems

The findings that ANT inhibitors and ligands can influence the permeability transition suggested ANT involvement in this phenomenon. However, it was not clear whether ANT could only play a modulatory role or whether it could act as the pore. The answer to this question step-by-step came from experiments with reconstituted systems. Reinhard Krämer’s group (1990) demonstrated that the aspartate/glutamate carrier and ANT modified by SH-reagents could be converted from an obligate exchange mechanism to a unidirectional transport mode, suggesting channel-like activity of the aspartate/glutamate carrier and ANT [39,40]. Using electrophysiological patch-clamp techniques, this group also demonstrated that a P_i_ carrier from *Saccharomyces cerevisiae* mitochondria could behave as an ion channel, although with relatively low conductance, ranging from 25 to 40 pS in the presence of Ca^2+^ and Mg^2+^ [41].

In experiments with ANT reconstituted into a planar bilayer phospholipid membrane, Vladimir Skulachev’s group (1994) detected large ion currents following application of the SH-reagent mersalyl [42]. The channel appeared non-selective with multiple sub-levels of conductance ranging from 200 pS up to 1.5 nS. This study demonstrated the long-suspected ability of modified ANT to operate as a pore. However, the possibility of Ca^2+^-induced transformation of ANT into the pore was not demonstrated in this study.

The possibility of Ca^2+^-induced transformation of ANT into the pore has been demonstrated in our studies with purified bovine heart ANT reconstituted into giant proteoliposomes [43]. In these experiments, we discovered a Ca^2+^-dependent, large non-selective channel with multiple sub-states of conductance ranging from 300 to 600 pS in symmetrical 100 mM KCl. Based on its behavior, the ANT-associated channel could be easily distinguished from the porin channel (VDAC). The ANT channel opening required high Ca^2+^ that we proposed to bind to cardiolipin molecules, which are tightly associated with the ANT [44]. The ANT channel depended on pH and closed at pH 5.2. In addition, the ANT channel could be partly inhibited by bongkrekic acid and completely inhibited by a combination of bongkrekic acid and ADP. Carboxyatractyloside, as well as cyclosporin A, were without effect. The latter is not surprising because cyclophilin D was not present in these proteoliposomes. These experiments were based on an early idea that ANT operated as a gated channel [22] and showed that ANT indeed could be converted into a large non-selective channel by exposure to high Ca^2+^.

In the next study, we reconstituted recombinant ANT from *Neurospora crassa* into giant proteoliposomes and found a large non-selective channel with multiple conductance sub-levels and an open state conductance of 500–700 pS in symmetrical 100 mM KCl [45]. The ANT channel required Ca^2+^ for activation and could be inhibited by ADP and bongkrekic acid. When purified cyclophilin from *Neurospora crassa* (a single form of cyclophilin is present in both cytosol and mitochondria [46]) was added, it stabilized the ANT channel in the open state. CsA abolished the effect of cyclophilin. Thus, these experiments filled a critical gap in our previous study by showing the stabilizing effect of cyclophilin on the ANT channel open state and CsA-induced inhibition. Overall, the properties of the ANT channel strongly resembled the PTP. This and other observations regarding ANT involvement in the permeability transition suggested that the ANT could be a key conducting component of the PTP. This notion is supported by ANT’s crystal structure [47] that revealed a 30 Å deep and 20 Å wide depression in the carrier molecule. When the ANT is stabilized in the *c*-conformation by carboxyatractyloside, this depression appeared to be open to the cytosolic side, providing a structural basis for pore formation.

In 1996, Dieter Brdiczka’s group reconstituted an ANT-porin-hexokinase (or creatine kinase) complex in a planar bilayer phospholipid membrane and detected a large channel with a conductance of 6 nS [48]. The ANT-porin-kinase complex was also reconstituted into phospholipid liposomes loaded with malate or ATP. Ca^2+^ induced malate and ATP release, whereas N-methylVal-4-cyclosporin, a derivative of CsA [35], inhibited the release of solutes. Based on these observations, the authors concluded that the ANT-porin-kinase complex constitutes the PTP [48]. Although this study’s results, to some extent, resembled our findings [43], there was one significant distinction. The ANT-porin-kinase pore detected in electrophysiological experiments did not require Ca^2+^ to operate as a pore, suggesting that porin interaction with ANT alters ANT channel behavior (although in metabolite efflux experiments, Ca^2+^ was necessary to stimulate malate and ATP leakage) [48].

In the subsequent study, Brdiczka’s group (1998) showed that only ANT-porin-hexokinase but not ANT-porin-creatine kinase complex could be stimulated by Ca^2+^ to release malate and ATP from proteoliposomes in an N-methylVal-4-cyclosporin-sensitive manner [49]. The authors also found mitochondrial CyD in the ANT-porin-hexokinase complex that could explain sensitivity to N-methylVal-4-cyclosporin. In the following study, Brdiczka’s group (1998) showed that ANT purified from rat heart mitochondria and reconstituted into asolectin/cardiolipin vesicles loaded with ATP and malate allowed release of the trapped compounds in response to increasing concentrations of Ca^2+^ [50]. This Ca^2+^-induced solute leakage was not sensitive to N-methylVal-4-cyclosporin, but ADP inhibited the leakage. On the other hand, atractyloside and HgCl_2_ stimulated the release of solutes from ANT-reconstituted liposomes. These results strongly suggested that ANT itself can adopt a pore-like conformation under conditions known to induce the permeability transition in mitochondria [50]. Soon after these studies, Guido Kroemer’s group (2000) reported that consistent with our observations [43], ANT reconstituted in the planar bilayer lipid membrane did not mediate ion currents unless Ca^2+^ or atractyloside are applied [51]. Both Ca^2+^ and atractyloside stimulated ANT channel activity, reaching in symmetrical 100 mM KCl conductance of 250 and 30 pS, respectively. Taken together, numerous experimental findings obtained in different laboratories with isolated mitochondria and reconstituted systems converged at the conclusion that ANT can be converted into a pore by Ca^2+^ or other factors promoting the permeability transition, and therefore, modified ANT could be a conducting component of the PTP.

## 6. Some Discrepancies in Studies of ANT as a Pore

Although the ANT hypothesis received significant experimental support from many independent investigators, some studies produced results that seem difficult to reconcile with ANT’s key role in the permeability transition pore. In early studies, Hunter and Haworth (1979) found the ADP inhibitory effect on the PTP is mediated by ADP acting at two different binding sites [8]. One of these sites appeared to be high affinity and atractyloside-sensitive, belonging to the ANT, while the other is a low affinity and carboxyatractyloside-insensitive site of unknown molecular identity. Novgorodov et al. (1991–1992) showed that in liver and heart mitochondria, the inhibitory effect of CsA was augmented by low concentrations of ADP in a carboxyatractyloside-sensitive manner [52,53]. In these studies, higher concentrations of ADP in combination with CsA inhibited the pore even in the presence of carboxyatractyloside. Based on these findings, the authors proposed that the pore is probably not formed directly by the ANT, and that another ADP-binding protein with low affinity to ADP could be involved in the pore induction/activation. Gizatullina et al. (2005) also reported that high ADP (2 mM) could inhibit the pore in the presence of carboxyatractyloside [54]. In this study, the authors provided some evidence that the low-affinity regulatory ADP binding site is located on the inner membrane’s outer side. Halestrap’s group (1997) reported that, in liver mitochondria, the K_i_ for the inhibitory effect of ADP in the absence and presence of carboxyatractyloside is 1.9 ± 0.4 µM and 27.2 ± 5.2 µM, respectively [55]. The authors speculated that this might reflect the existence of two ADP binding sites, one of high and the other of low affinity on the ANT, of which the former is sensitive and the latter insensitive to carboxyatractyloside. However, carboxyatractyloside-insensitive ADP inhibition of the PTP may occur due to ADP binding to a protein other than the ANT, such as to mitochondrial Ca^2+^ uniporter, which also interacts with ATP and ADP [56] or due to ADP binding to F_0_F_1_-ATP synthase, which was recently implicated in PTP induction [57,58,59].

## 7. Genetic Manipulations of ANT Expression and the Role of ANT in PTP

The use of genetic manipulation techniques offered exciting new opportunities in studies of the molecular composition of the PTP. Genetic ablation of ANT was used as a test for its involvement in PTP formation. Using this approach, Doug Wallace’s group (2004) generated mice lacking both ANT1 and ANT2 in liver mitochondria [60]. In a series of well-designed experiments, the authors demonstrated that the equal Ca^2+^ loads that induced the PTP in mitochondria from wild-type mice could not induce PTP in mitochondria from genetically modified mice, lacking ANT1 and ANT2. This apparently indicated the key role of ANT in PTP induction. However, the authors came to the opposite conclusion since a 3-fold increase in Ca^2+^ load did cause PTP induction in mitochondria of ANT-deficient mice. Consequently, the authors concluded that ANT does not play an essential role in PTP. By the way, there is a report that skeletal muscle mitochondria lacking ANT require an 8-times larger Ca^2+^ load to induce the permeability transition [61]. Such greatly increased ANT-deficient mitochondria resistance to Ca^2+^ hardly be considered evidence for a “non-essential” role of ANT in PTP. Correspondingly, the conclusion about the “non-essential” role of ANT in PTP seems misleading. In our opinion, a significant increase in the amount of Ca^2+^ required for PTP induction in mitochondria from ANT-deficient mice does not disprove an essential role of ANT in PTP, but rather suggests the existence of additional mechanisms involved in PTP formation.

## 8. Alternative Mechanisms of Conducting Pore Formation in the PTP

Indeed, over the next ten years, new hypotheses regarding the composition of the PTP have emerged. Andrew Halestrap (2008) proposed that a P_i_ transporter in the inner mitochondrial membrane could be a conducting pathway in the PTP [62]. This is in line with Reinhard Krämer’s group’s findings that a P_i_ transporter can be converted into a channel-like structure following treatment with mercurials [63]. In another recent study, investigators provided evidence suggesting that the *c*-subunit of the F_0_ complex in F_0_F_1_-ATP synthase could be involved in PTP operation [57]. Another remarkable study by Elizabeth Jonas’ group provided strong evidence that the *c*-subunit ring of F_0_F_1_-ATP synthase may constitute the PTP [59]. However, atomistic simulations suggest that the *c*-subunit ring of the F_0_F_1_-ATP synthase cannot be the PTP [64].

Paolo Bernardi’s group proposed an alternative view of the role of F_0_F_1_-ATP synthase in PTP formation. This group demonstrated that dimers of F_0_F_1_-ATP synthase could form a large channel in the planar lipid bilayer [58]. This channel was Ca^2+^-dependent, could be inhibited by ADP and Mg^2+^, and was insensitive to CsA and ANT inhibitors. To some extent, this channel resembled the “megachannel” described in early studies and attributed to the PTP [65,66,67]. Further, it was proposed that this PTP-like channel is formed at the membrane interface between two adjacent F_0_ complexes [6]. Despite significant evidence in favor of the F_0_F_1_-ATP synthase hypothesis, genetic manipulations with F_0_F_1_-ATP synthase, which seem to be incompatible with the pore formation, failed to completely prevent PTP induction [68,69,70].

## 9. Reinvigoration of ANT Hypothesis

Two recent studies renewed the interest in ANT involvement in Ca^2+^-induced permeability transition and might resolve the existing discrepancies. Karch et al. (2019), using liver mitochondria from *Ant1*, *Ant2*, and *Ant4* deficient mice and Ca^2+^ uptake capacity and light scattering assays, demonstrated significant inhibition of PTP induction [71]. Much more Ca^2+^ was required for induction of the PTP in mitochondria from *Ant*-triple-knockout mice compared to mitochondria from wild-type animals. Additional deletion of CyD further increased resistance of mitochondria to PTP induction. In electrophysiological patch-clamp experiments with mouse embryonic fibroblasts (MEF) mitoplasts from *Ant*-triple-knockout mice, the authors found an almost complete lack of pore-like activity. The rare pores detected in these experiments were insensitive to ADP. The authors proposed that the ANT family is the primary inner-membrane channel-forming component of the MPTP in MEFs and concluded that PTP consists of ANT and an unknown component requiring CyD [71]. These results, however, do not rule out a contribution of other components, such as a mitochondrial Pi carrier or F_0_F_1_-ATP synthase, to PTP formation.

Evgeny Pavlov’s group performed another interesting study. The authors used F_0_F_1_-ATP synthase *c*-subunit knockout mitochondria from HAP1-A12 cells to investigate the role of the *c*-subunit in the PTP [72]. Using an electrophysiological patch-clamp approach, the authors found that deletion of *c*-subunit eliminates the classic PTP conductance of approximately 1.5 nS. Simultaneously, in *c*-subunit knockout cells, the authors detected another channel with conductance around 300 pS, which was sensitive to cyclosporin A and bongkrekic acid, an inhibitor of ANT. The authors concluded that while the *c*-subunit of F_0_F_1_-ATP synthase might be the primary contributor to PTP activity, other proteins, e.g., ANT, could also be involved in Ca^2+^-induced PTP formation. This may explain why genetic manipulation leading to inactivation or deleting F_0_F_1_-ATP synthase components may not completely prevent permeability transition [68,69,70].

Interestingly, mitochondria from *c*-subunit knockout cells more easily undergo permeability transition compared to mitochondria from wild-type cells [72], suggesting that permeability transition linked to the opening of the ANT-associated pore may require less Ca^2+^, has a higher likelihood of induction, and precede induction of the pore associated with F_0_F_1_-ATP synthase or other protein/protein complex. This is perfectly consistent with a significantly increased amount of Ca^2+^ required for PTP induction in mitochondria from *Ant1*-knockout mice [61], *Ant1-* and *Ant2*-double-knockout mice [60], and *Ant*-triple-knockout mice [71]. Overall, identifying a specific protein such as ANT or a protein complex such as F_0_F_1_ ATP synthase as a “bona fide” PTP is most likely meaningless because different proteins/protein complexes may increase the permeability of the inner mitochondrial membrane. Therefore, the term PTP may comprise different molecular entities involved in the phenomenon of the permeability transition.

## 10. Conclusions

Taken together, none of the existing observations refute a crucial role of ANT in the permeability transition. Moreover, none of these data rule out a pore-forming function of ANT in the PTP. However, the recent data strongly suggest a multiplicity of the mechanisms underlying the permeability transition. This idea is not new and has been introduced by Mario Zoratti and colleagues 15 years ago in their excellent review paper on the PTP [73] and by Martin Klingenberg, a world-renowned expert on ANT, in his excellent review paper on ANT [74]. The “exclusion method”, when the investigators genetically delete a certain protein in an attempt to determine its involvement in a specific process, may not be applicable in the case of PTP investigation. Assuming a possible variety of mechanisms and many molecular entities underlying the PTP, removing a single pore-forming molecular component contributing to PTP may not eliminate the whole PTP phenomenon. However, it may rather change some of its properties, such as increasing Ca^2+^ load or prolonging the time necessary for PTP formation. It seems conceivable that accepting the idea regarding the multiplicity of the permeability transition mechanisms could be a key to answering many questions about structure, function, and the pharmacological profile of the PTP. Consequently, accepting the idea that multiple mechanisms underlie the permeability transition may help to understand the complex nature of this phenomenon better and resolve many controversial issues regarding the molecular identity of the PTP.
